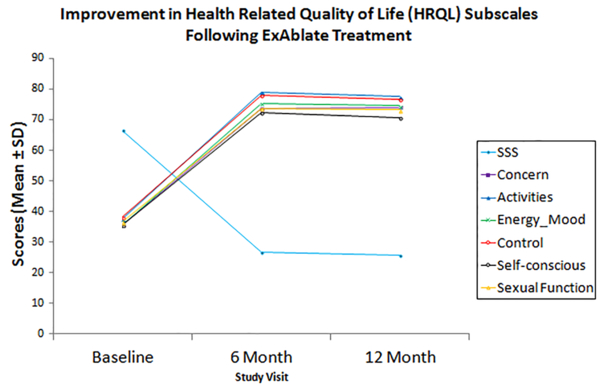# MR guided focused US for treatment of uterine fibroids: symptom reduction in a multicenter trial using a novel treatment algorithm

**DOI:** 10.1186/2050-5736-3-S1-O91

**Published:** 2015-06-30

**Authors:** Nelly Tan, David Lu, Steven Raman, Simin Bahrami

**Affiliations:** 1 University of California at Los Angeles, Los Angeles, California, United States

## Background/introduction

Enhanced sonication (ES) is a newer technique to ablate symptomatic uterine fibroids. ES uses nearly twice the amount of energy and is able ablate nearly twice the region of interest compared to standard sonications. As a result, more volume of uterine fibroids are able to be treated within the same amount of time. The objective of the multi-center trial was to evaluate the clinical efficacy of enhanced sonication.

## Methods

A HIPAA-complaint, IRB-approved prospective single-arm multi-center trial study of women consecutively evaluated at seven tertiary care institutions in the United States of women with symptomatic uterine fibroids was performed. The study was approved by the institutional review board at seven tertiary care centers in the United States between Jan 2010 and Mar-2013. This study was under a post-PMA approval (open label), single-arm, multicenter study using the ExAblate 2000 system. Patients were followed for safety at one week and one month post-treatment. For effectiveness patients had to consent again to be followed for 3 years. Safety was measured by the incidence, severity and duration of adverse events; effectiveness was measured by immediate post-treatment non-perfused ratio, SSS-UFS-QOL score as compared to baseline and retreatment rate. We performed paired t-test to evaluate the difference in UFS-QOL pre and post treatment. Statistics were considered significant at p = 0.05.

## Results and conclusions

Out of 245 screened patients 115 patients underwent 164 treatment sessions (54 patients had a second treatment.) Of these, 100 agreed to be followed up. Patients were an average age of 44 and average BMI of 24.9. Of 115, 71 were Caucasian, 13 African American, 8 Asian, and 8 others. 58 patients had a single fibroid, 28 patients had between 2 to 4 fibroids, and 14 patients had 5 or more fibroids. The total fibroids volume per patient was in average 235cc ± 220 (range 10-1300). The average nonperfused ratio measured immediately after the treatment was 65% ±23% (range 6%-100%). Baseline SSS for the 100 patients was 66.7±15.7 (range 28-100). At 6 months, in 91 patients who followed up, the SSS decreased to 26.8±16.2 (range 0-75). There were significant improvements in the UFS-QOL score overall and the sub-scales including symptom severity score (66.4 *vs*. 25.5, p<0.01), concern (35.7 v. 74.0, p<0.01), activities (37.61 *vs*. 77.5, p<0.01), energy (35.7 v 74.4, p<0.01), control (38.2 v. 76.6, p<0.01), self-consciousness (35.5 v 70.6, p<0.01), sexual function (36.5 v. 73.3, p<0.01) and health-related quality of life (36.6 v. 75, p<0.01) between baseline and 12 month post treatment, respectively. The number of patients who underwent alternative treatments at 12 months was 9 (9%). There were no major complications. Minor complications occurred in <5% of the patients. Those that lasted longer than 7 days were one skin burn, patient reported fatigue, one complaint of abdominal cramping, one of perineal pain, and one with leg pain for two months.

Conclusion: In this multicenter cohort, sustained symptom relief was possible up to 12 months with the addition of enhanced sonicatons to standard sonication during MR guided HIFU.

**Figure 1:**